# Community-associated Methicillin-resistant *Staphylococcus aureus*, Switzerland

**DOI:** 10.3201/eid1106.041308

**Published:** 2005-06

**Authors:** Stephan Harbarth, Patrice François, Jacques Schrenzel, Carolina Fankhauser-Rodriguez, Stephane Hugonnet, Thibaud Koessler, Antoine Huyghe, Didier Pittet

**Affiliations:** * University of Geneva Hospitals, Geneva, Switzerland

**Keywords:** Staphylococcus aureus, methicillin resistance, prevalence, Switzerland, community, risk factor

## Abstract

Two case-control studies evaluated the prevalence of community-associated methicillin-resistant *Staphylococcus aureus* (CA-MRSA) carriage at hospital admission and characteristics of patients with CA-MRSA. Among 14,253 patients, CA-MRSA prevalence was 0.9/1,000 admissions. Although 5 CA-MRSA isolates contained Panton-Valentine leukocidin, only 1 patient had a previous skin infection. No easily modifiable risk factor for CA-MRSA was identified.

Recently, new strains of community-associated methicillin-resistant *Staphylococcus aureus* (CA-MRSA), which cause soft tissue infections in healthy people, have been detected worldwide (1). The unique molecular feature of these CA-MRSA strains consists of 2 particular genetic elements, the type IV staphylococcal cassette chromosome (SCC) *mec* element and a virulence gene encoding a leukocyte-killing toxin called Panton-Valentine leukocidin (PVL), not found in hospital-acquired MRSA isolates (1).

Risk factors for CA-MRSA carriage are incompletely understood. Although antimicrobial drug use is well recognized as risk factor for hospital-acquired MRSA (2), results of previous investigations have been inconsistent regarding the association between previous antimicrobial drug use and acquisition of CA-MRSA (3,4). Recently, 2 studies from North America have suggested that recent antimicrobial drug use plays a role in CA-MRSA colonization (5,6).

Few systematic studies have assessed the epidemiology of CA-MRSA in Europe (7). Determining the epidemiology of CA-MRSA could help develop control measures and guide clinicians in identifying patients at high risk for CA-MRSA. Therefore, our prospective investigation sought to 1) determine the prevalence of CA-MRSA on hospital admission, 2) examine characteristics of patients carrying CA-MRSA, 3) test the hypothesis that previous antimicrobial drug exposure is associated with CA-MRSA carriage, and 4) evaluate the genetic diversity of CA-MRSA strains.

## The Study

Details of this prospective, observational study have been presented elsewhere (8). In brief, the study population consisted of 14,253 patients who were screened for MRSA carriage on admission to the Geneva University Hospitals between January 20, 2003, and August 31, 2003. Of these patients, 12,072 (85%) were hospitalized in the adult wards, 102 (1%) in pediatric wards, and 361 (2%) in psychiatric wards; 1,718 (12%) were seen in the emergency room and were discharged within 24 hours. MRSA screening was performed by nasal and inguinal swab samples, and cultures of specimens from other sites were performed when clinically indicated. A person fulfilled the CA-MRSA case definition if 1) the person had an MRSA isolate that yielded a SCC*mec* type different from the prevailing hospital-associated strain in the Geneva region (SCC*mec* type I [9]) and 2) the person had not been hospitalized within the last 3 years (3).

We performed 2 case-control studies. The first control group comprised all patients with MRSA carriage identified on admission who did not fulfill our case-definition of CA-MRSA. If a patient was admitted more than once during the study period, only the first admission was included in this analysis. The second control group consisted of a group of randomly selected MRSA-negative patients. The following potential risk factors for CA-MRSA carriage were documented: age, sex, origin of patient, coexisting conditions, severity of underlying illness, functional status, patient's prior location, presence of skin lesions, and antimicrobial drug use within the past 6 months.

MRSA was identified according to NCCLS guidelines (10). Typing of SCC*mec* elements and detection of PVL genes were carried out as described (9). A novel multiplex polymerase chain reaction (PCR)-based assay was used for rapid genotyping of *S*. *aureus* isolates (11). This assay is based on variable-number tandem repeat typing (12) and has been modified to allow high throughput and automated analysis. In addition, 13 CA-MRSA isolates, 2 hospital-acquired MRSA isolates, and 2 references strains from the United States were genotyped by multilocus sequence typing (MLST) (13). PCR products were sequenced with an ABI Prism 3100 DNA sequencer (Applied Biosystems, Foster City, CA, USA). Allele numbers were assigned according to the program available from the MLST Web site (http://www.mlst.net).

We used the Student *t* test to compare continuous variables and the chi-square test or Fisher exact test to compare proportions. Univariate comparisons were performed to determine characteristics of CA-MRSA patients. Data were analyzed with STATA, version 8.0 (StataCorp, College Station, TX, USA).

During January through August 2003, 428 of 14,253 screened patients were discovered to be MRSA carriers on admission (prevalence 3.0%). Most MRSA isolates belonged to the type I cassette (n = 371, 26/1,000 admissions). MRSA SCC*mec* type IV was recovered in 46 patients (3.2/1,000 admissions), whereas types II, III, and V were only rarely identified (n = 11). Thirty-seven of 46 patients (80%) with SCC*mec* type IV isolates had previous contact with the healthcare system, in particular with hospitals in neighboring France.

Thirteen patients fulfilled our case definition for having CA-MRSA (prevalence 0.9/1,000 admissions). The prevalence of CA-MRSA varied according to the hospital sector: it was highest in pediatric patients (9.8/1,000), followed by adult outpatients staying <24 hours (1.7/1,000) and adult inpatients (0.7/1,000). Important features of the 13 CA-MRSA cases are shown in the Table. Six CA-MRSA patients lived outside Switzerland: Kosovo (n = 2), France (n = 1), Senegal (n = 1), Madagascar (n = 1), and Libya (n = 1).

Ten CA-MRSA isolates harbored the SCC*mec* type IV, 2 the type V, and 1 the type II element (Figure). All CA-MRSA isolates were susceptible to trimethoprim-sulfamethoxazole, clindamycin, and vancomycin. Two isolates (SCC*mec* type V) were resistant to gentamicin; 2 other isolates (SCC*mec* type IV) showed resistance to fluoroquinolones; and 1 (SCC*mec* type II) was resistant to macrolides. Although 5 (38%) of 13 CA-MRSA isolates possessed the *pvl* gene, only 1 patient had a skin infection on admission. The other 4 patients had no history of infection.

**Figure:**
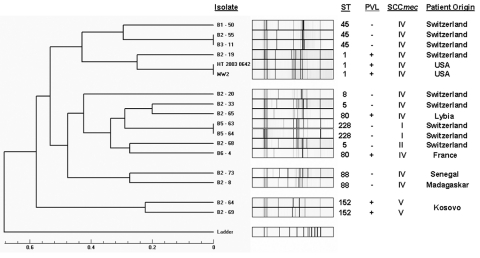
Analysis of genotyping patterns, multilocus sequence typing (ST) results, presence of Panton-Valentine leukocidin (PVL), staphylococcal cassette chromosome *mec* (SCC*mec*) type, and country of patient origin of 13 community-associated, methicillin-resistant *Staphylococcus aureus* isolates (CA-MRSA). The dendrogram illustrates the genetic relatedness of the 13 CA-MRSA in comparison to 1) 2 nosocomial MRSA isolates representing the prevailing endemic strain in the Geneva healthcare setting (strains B5-63, B5-64) and to 2) profiles obtained for strains MW2 and HT20030642 from the United States, 2 closely related CA-MRSA isolates used as controls.

Genetic analysis combining genotyping and MLST showed substantial diversity among CA-MRSA isolates (Figure). In particular, they were not related to the nosocomial strain endemic in the Geneva hospital setting (strains B5-63 and B5-64). Dendrogram analysis identified 2 closely related CA-MRSA isolates from patients living in Geneva (strains B2-55 and B3-11) who had never been hospitalized and had no apparent epidemiologic link. The pattern of 1 CA-MRSA isolate (B2-19), from a 38-year-old Geneva woman who used injection drugs, was related to the MW2 strain from North Dakota.

MLST of CA-MRSA isolates identified 7 sequence types (ST-1, -5, -8, -45, -80, -88, and -152), belonging to patients from different geographic origins (Figure). Five strains represented 2 distinct ST clones (ST-45 and ST-152) previously described in northern Europe and Israel; 1 isolate was related to the prototype CA-MRSA strain MW2 (ST-1); and 2 isolates corresponded to a clone reported from Asia (ST-88). The largest cluster contained strains previously described from several continents, including French clone ST-8, Mediterranean clone ST-80, and the international, so-called pediatric clone ST-5 (SCC*mec* type IV).

Patients with CA-MRSA differed in several ways from those who carried healthcare-associated MRSA (n = 346) and those who were free of MRSA on admission (n = 1,542, Table). Patients with CA-MRSA were younger and more likely to have a permanent residency outside Switzerland. Compared to non-MRSA controls, no significant differences were noted in previous outpatient antimicrobial drug use and presence of coexisting conditions (Table). By contrast, CA-MRSA patients had fewer coexisting conditions and less prior exposure to antimicrobial drugs than patients with healthcare-associated MRSA. The presence of skin lesions on admission was not predictive of CA-MRSA carriage.

**Table:** Characteristics of patients with community-associated methicillin-resistant *Staphylococcus aureus* (CA-MRSA), compared to those of patients without MRSA and patients with healthcare-associated MRSA

Characteristic	Patients with CA-MRSA (n = 13) (%)	Patients without MRSA (n = 1,542) (%)	p value*	Patients with healthcare-associated MRSA (n = 346) (%)	p value†
Mean age (y ± SD)	45 ± 25	70 ± 18	<0.001	75 ± 15	<0.001
Male sex	6 (46)	682 (44)	1.0	199 (58)	0.57
Residency outside Switzerland	6 (46)	10 (1)	<0.001	8 (2)	<0.001
Healthcare worker	0 (0)	31 (2)	1.0	3 (1)	1.0
Emergency admission	9 (69)	724 (48)	0.17	166 (48)	0.16
Severity of disease					
Presence of ≥1 coexisting condition	8 (62)	1,082 (70)	0.55	289 (84)	0.05
Rapidly or ultimately fatal disease	0	277 (18)	0.11	95 (27)	0.02
Complete dependence for daily activities	0	114 (7)	0.62	57 (16)	0.24
Antimicrobial drug exposure					
Previous exposure (<6 mo)	1 (8)	318 (21)	0.49	197 (57)	<0.001
Current use at admission	3 (23)	148 (10)	0.13	41 (12)	0.21
Presence at admission of indwelling urinary catheter	0	73 (5)	1.0	64 (19)	0.14
Open skin lesions	2 (15)	100 (6)	0.21	65 (19)	1.0

*Community-associated MRSA case-patients versus non-MRSA controls. †Community-associated MRSA case-patients versus controls with healthcare-associated MRSA strains (SCC*mec* type I isolates).

## Conclusions

This study provides information about the epidemiology of CA-MRSA on admission to the largest hospital in Switzerland. It showed 1) a low prevalence of CA-MRSA, 2) a reservoir of asymptomatic persons colonized with PVL-containing CA-MRSA strains, 3) a high degree of CA-MRSA diversity, and 4) no readily modifiable risk factor for CAMRSA carriage.

Several investigators have recently attempted to describe the epidemiology of CA-MRSA more precisely (5,14). These study findings are not consistent since they were conducted in different settings and used various case definitions (3). Typically, most studies used an epidemiologic case definition that excluded patients with recent healthcare system contact in whom MRSA was detected within 48 hours after hospital admission (15). This type of study, however, cannot prove that MRSA acquisition was unrelated to healthcare system contact. Therefore, how to define true community-acquired MRSA remains controversial. Although we added a molecular component to increase the validity of our case definition, we used the more conservative term "community-associated" MRSA, since we cannot exclude the fact that CA-MRSA casepatients may have previously been in contact with outpatient care or hospitalized family members.

Described risk factors associated with CA-MRSA infection include intravenous drug use, military training, jail exposure, team sport activities, homosexuality, low socioeconomic class, and being member of a "closed population" such as Native Americans and Australian aborigines (5,6). Two recent analyses found an increased risk in patients exposed to antimicrobial drugs (5,6). Our study did not confirm this hypothesis, making it unlikely that antimicrobial drug control measures will substantially decrease transmission of CA-MRSA.

Control of CA-MRSA remains a challenge, since transmission is linked to migration and travel (16). Our study showed that the ratio of 4:1 between colonization and infection with CA-MRSA possessing the *pvl* gene was larger than previously thought. Restricting surveillance to infected carriers will underestimate the prevalence of PVL-producing CA-MRSA. Thus, CA-MRSA surveillance should not rely on clinical specimens alone.

Several study limitations merit consideration. First, we may have underestimated CA-MRSA prevalence since our study was not truly population-based. Second, we may have misclassified CA-MRSA cases. Thorough review of medical charts minimized this potential bias. Finally, we were not able to elucidate the route of transmission for those CA-MRSA carriers living in the Geneva community. MLST results suggest that nonendemic hospital strains may already circulate in the Geneva community.

In summary, the prevalence of CA-MRSA remains relatively low in our community. Yet migration will likely increase CA-MRSA carriage in the near future. This development will influence clinical practice by changing the choice of empiric antimicrobial drug therapy for severe skin and soft tissue infections.
